# Changes in Biochemical Composition and Energy Reserves Associated With Sexual Maturation of *Octopus maya*

**DOI:** 10.3389/fphys.2020.00022

**Published:** 2020-02-04

**Authors:** Cristina Pascual, Honorio Cruz-Lopez, Maite Mascaró, Pedro Gallardo, Ariadna Sánchez, Pedro Domingues, Carlos Rosas

**Affiliations:** ^1^Unidad Multidisciplinaria de Docencia e Investigación, Facultad de Ciencias, Universidad Nacional Autónoma de México, Hunucmá, Mexico; ^2^Laboratorio Nacional de Resiliencia Costera (LANRESC), CONACYT, Mexico City, Mexico; ^3^Posgrado en Oceanografía Costera, Universidad Autónoma de Baja California, Carretera Transpeninsular Ensenada, Ensenada, Mexico; ^4^Instituto Español de Oceanografía – Centro Oceanográfico de Vigo, Vigo, Spain

**Keywords:** body size, season, energy reserves, hemolymph, Octopus maya

## Abstract

Climate conditions are related to changes in the biochemical composition of several tissues and associated to the processes of growth and sexual development in cephalopods. The biochemical composition (protein, glucose, cholesterol, acylglycerides) and hemocytes of the hemolymph, the hepatosomatic and gonadosomatic indices, and the reserves of the gonad, hepatopancreas and muscle (lipids, glycogen, and caloric value of muscle) of *Octopus maya* were determined and related to sex and season. A total of 154 wild animals were used (≈50 caught per season) and the multivariate analysis of the biochemical indicators of the tissues allowed following the variations during winter, dry and rainy season. The permutational MANOVA showed that both sex and season contributed significantly to variations in metabolites and energy reserves. However, the non-significant interaction term indicated that the biochemical composition changed with the seasons in a similar way and regardless of sex. The pattern observed in metabolites and reserves indicates a variation associated with growth and the reproductive peak, but may also reflect a physiological response to seawater temperature. The present study provides reference values for several physiological indicators in *O. maya* that may be useful for programs monitoring wild populations, as well as to design diets and management protocols to produce octopus under controlled conditions.

## Introduction

Cephalopods are thought to be the most evolved group among marine molluscs (O’Dor and Webber, 1986). Along with fish, cephalopods comprise a significant part of the ocean biomass (Grisley and Boyle, 1985, 1988), and their position in the food chain grants them a relevant place in energy transfer among different trophic levels (Jackson et al., 1998). Important commercial species such as fish and lobsters among their prey, whereas their predators are marine mammals, fish, and birds (Guerra, 1992; Hernández-García, 1995; Rocha and Vega, 2003). The red octopus (*Octopus maya*) is endemic to southeast Mexico and is one of its most important fishery resources (Voss and Solís-Ramírez, 1966; Solís-Ramírez and Chávez, 1986; Solís-Ramírez, 1988). Red octopus fishery begun its development in 1949 (Solís-Ramírez et al., 1997) and its commercial value has driven its growth; during the period 1998–2016 national catches varied between 9,000 and 35,000 t/year (Markaida et al., 2017).

*Octopus maya* has a semelparous reproductive strategy, with females reproducing only once and dying shortly after the eggs hatch, as is common in many cephalopod species (Cortéz et al., 1995; Zúñiga-Romero et al., 1995; Rocha et al., 2001). In captivity, *O. maya* life span is between 8 and 12 months at 25–30°C (Van Heukelem, 1983; Hanlon and Forsythe, 1985). The study of the population structure and maturation indicates that *O. maya* grows and matures during the fishing season (August to December). Adults show functional maturity during the winter storm season (November to February), when seawater temperature oscillates around 25°C (Angeles-González et al., 2017). Spent females predominate in January and February, revealing a year-long life cycle (Markaida et al., 2017). Embryonic development lasts 40 to 50 days, depending on temperature stability under cultured conditions (Rosas et al., 2014), and 50 to 65 days in natural environments (Solís-Ramírez, 1967). Voss and Solís-Ramírez (1966), reported that *O. maya* produces the largest eggs among octopodids, measuring up to 17 mm. Direct development and high adaptability to captivity make *O. maya* one of the most promising cephalopod species for commercial aquaculture (Boletzky and Hanlon, 1983; Tercero et al., 2015).

*Octopus maya* inhabits shallow waters adjacent to the continental shelf of the Yucatan Peninsula (states of Campeche, Yucatan, and Quintana Roo). This species is often found in areas covered by sea grass (*Thallassia testudinum*), gastropod empty shells and coral fragments (Voss and Solís-Ramírez, 1966). At the Yucatan Peninsula, a transition site between the Caribbean Sea and the Gulf of Mexico, three climatic seasons can be identified: (1) the winter season (“nortes;” November to February), with low atmospheric temperatures (mean 23°C) and moderate precipitation (40–150 mm/month); (2) the dry season (March to May), with atmospheric temperatures around 36–38°C and low precipitation (0–30 mm/month); and (3) the rainy season (June to October), with atmospheric temperatures higher than 38°C and precipitation above 220 mm/month, even higher than 350 mm/month if hurricanes occur (Herrera-Silveira et al., 1998).

Cephalopods are highly sensitive to environmental variations, which directly impact their abundance and lifecycle (Pecl et al., 2004; Leporati et al., 2008; Pierce et al., 2008), both in natural populations (Cortéz et al., 1999), and under cultured conditions (Hanlon and Forsythe, 1985; DeRusha et al., 1987). Clarke et al. (1994) reported *Illex argentinus* biochemical composition associated to energy demands during growth and sexual maturation. Otero et al. (2007) observed that the weight of the digestive gland in *Octopus vulgaris* females increased proportionally to the maturity index. Likewise, De Moreno et al. (1998) found greater biochemical variations in female *Illex argentinus* probably associated with oogenesis.

Lipid energy reserves have an important role in the physiology of marine animals. They provide food supply for oocytes, ensuring the viability of the larvae (Giese, 1966; Giese et al., 1967). Several studies on this topic have been conducted in cephalopods. Rosa et al. (2002, 2004, 2005) studied changes in the biochemical composition of different tissues in several cephalopod species with diverse life and sexual maturation strategies. They found that the biochemical composition of the digestive gland and muscle is related to feeding, food availability, spawning and incubation. Nutritional status influences the time and intensity of the reproductive events (Sibly and Calow, 1986) and breeding frequently depletes animals energy reserves. Pollero and Iribarne (1988) studied the biochemical changes in different tissues during the reproductive cycle of *Octopus tehuelchus.* They reported that females undergo greater biochemical changes: lipid contents change primarily in gonadal tissue, increasing during sexual maturation and decreasing during the incubation period.

Reserves have a strong influence on sexual maturation, leading to nutrient mobilization and accumulation. A better understanding of the tissues biochemical composition is crucial to assess population health status and energy flow in ecosystems. Climatic seasons have been found to have a marked effect on tissues biochemical composition in other molluscan species and are associated with sexual development (Gámez-Meza et al., 1999; Berthelin et al., 2000; Orban et al., 2002). In this context, the present study aimed to characterize the biochemical composition of hemolymph, gonad, digestive gland, and muscle of wild *O. maya*, in order to assess seasonal variations and their relation to sexual maturation.

## Materials and Methods

### Octopus Sampling

Between May 2007 and February 2008, wild adult organisms were captured in coastal areas of Sisal, Yucatan, Mexico (21°09′N; 90°01′W). A total of 52 wild adult organisms were analyzed in three different climatic seasons and were assigned based on local seasonality: (1) the winter season (February); (2) the dry season (May) and (3) the rainy season (September). Six sampling days were performed per season, and three captures were conducted at 2 days intervals. Organisms were caught using the “gareteo” technique that allows catching the organisms without harming them (Solís-Ramírez, 1988). The physicochemical parameters of water (pH, temperature, salinity, and dissolved oxygen) were recorded at every capture using a Van Dorn bottle and a multi-sensor (Hatch), at an average depth of 8 meters. On board, organisms were placed inside a black tank with 1000 L of sea water in constant replacement by using a submersible pump. Transportation from the capture areas to the laboratory facilities took less than 2 h. Organisms were settled in individual green tanks (45 cm in diameter, 60 cm in depth, with 80 L of seawater) in a closed area with controlled photoperiod (12:12 light-dark), with constant aeration system and daily seawater flow exchange equivalent to 300% (28 ± 1°C, 37 ± 1 PSU, pH 8.1 ± 0.2). Samples were obtained the next day and organisms were not fed to avoid influencing the indicators of nutritional and physiological status.

### Tissues Samples and Plasma Analyses

Octopus were individually immersed in cold water (17°C below the maintenance system temperature) for 2 to 4 min (Cruz-López, 2010; Roumbedakis et al., 2017). When the respiratory rate and locomotor activity decreased, the animals were removed, and hemolymph was drawn from the aorta with a sterile catheter-cold connected to a 5 mL Falcon tube (Cruz-López, 2010). Approximately 0.5 mL of the hemolymph were transferred into tubes for hemocyanin and hemocytes analysis, and then stored (2–8°C) for 4 h. The samples were centrifuged at 800 × g for 5 min at 4°C to separate plasma, which was then used to assess the metabolites concentration in the plasma. Glucose, cholesterol, and acylglycerides concentrations were determined using enzymatic/colorimetric methods with commercial kits: Glucose Bayer (Sera Pak Plus B014509-01); acylglycerides (Sera Pak Plus, B01455101) and cholesterol (Bayer B01 4507-01). A total of 10 μL of plasma were added to 200 μL of the appropriate enzyme reagent for each sample. Protein concentrations were determined by the method of Bradford (1976), plasma was previously diluted with sterile water (400×) and 10 μL were mixed with 200 μL of Bradford reagent (Bio-Rad laboratories Cat. 500-0006). Absorbance values of all metabolites were recorded with a microplate reader (Benchmark Plus Bio-Rad). All samples were analyzed in triplicate and concentrations of metabolites (mg mL^–1^) were calculated using the standard curves.

The hemolymph was diluted into 1:100 using distilled water and measured at 335 nm (Thermo-Genesys 10 uv) and hemocyanin concentration (mM L^–1^) was determined using an extinction coefficient of 17.26 (Chen and Cheng, 1993a, b). Total hemocytes were counted in a Neubauer chamber from the hemolymph aliquot fixed with 4% formaldehyde in Alsever solution (115 mM C_6_H_12_O_6_, 30 mM Na_3_C_6_H_5_O_7_, 338 mM NaCl, 10 mM EDTA.Na_2_, pH 7.0) with a 1:3 dilution (Roumbedakis et al., 2017). Samples were kept at 2–8°C, for a maximum period of 10 days before analysis. All samples were tested in duplicate and expressed as cells/mm^3^.

### Tissues Biochemical Composition

Immediately after obtaining hemolymph, the octopus were euthanized by cerebral incision (Boyle, 1976; Fiorito et al., 2015). The animals were then weighted and dissected; the hepatopancreas and gonad were removed and weighted separately to estimate their proportion in relation to total weight (TW): hepatosomatic (HSI) and gonadosomatic (GSI) indices were calculated as follows: HSI = (DGW/TW) × 100; GSI = (GW/TW) × 100. Subsequently, those tissues and muscle were cut for lipids and glycogen analyses. Tissue sections were frozen in liquid nitrogen and stored at −40°C until analysis. Subsample of muscle were used for caloric analysis; the muscle was dried in the oven at 60°C until constant weight, and then placed in a calorimetric pump (Parr instruments).

Total lipids were assessed according to Folch et al. (1957); digestive gland, gonad and muscle tissues (0.5 g) were homogenized with a Teflon tip in 20 ml of chloroform/methanol solution (2:1) at 5000 × *g* for 2 min. The mixture was agitated for 15 min in an orbital shaker at ambient temperature. The homogenized was filtered (Whatman paper No 42). Samples were washed with water-methanol solution and placed in a separation funnel until two layers were formed. The chloroform-lipid fold was recovered and placed in a desiccation hood at 60°C with an air flow to complete evaporation. Glycogen in the tissues (digestive gland, gonad, and muscle) was extracted in trichloroacetic acid and determined by a reaction with sulfuric acid and phenol (Dubois et al., 1965). Approximately 0.4 g of the tissue was macerated with 200 μL of trichloroacetic acid (5%) and centrifuged for 6 min at 5000 × *g* (Eppendorf microcentrifuge 5415). Supernatant (100 μL) was pipetted into a tube and mixed with five volumes of 95% ethanol. Tubes were placed in an oven at 37°C for 3 h. After precipitation, the tubes were centrifuged again, the supernatant was discarded, and the tubes were drained. Finally, 200 μL of 5% phenol and 1 mL of sulfuric acid was added and samples were shaken with a vortex; subsequently 200 μL were placed in a microplate and measured (490 nm) using a microplate reader (Benchmark Plus Bio-Rad). The total amount of lipids and glycogen were analyzed in triplicate and expressed as mg g^–1^ of tissue.

### Statistical Analyses

A set of 14 descriptors measured in sexually mature *O. maya* were used to characterize changes in the biochemical composition and energy reserves of male and female octopus sampled in three different seasons (winter, dry, and rainy season). Descriptors included: hemocyanin concentration (Hemocyan, mM L^–1^), total hemocyte count (Hemocytes, cells mm^–3^), plasmatic proteins (Protein, mg mL^–1^), glucose (Glucose, mg mL^–1^), cholesterol (Cholest, mg mL^–1^), acylglycerides (Acylglycer, mg mL^–1^), lipids in gonads (LipidGonad, mg mL^–1^), hepatopancreas (LipidHepat, mg mL^–1^) and muscle (LipidMuscle, mg mL^– 1^), glycogen in hepatopancreas (GlucoHepat, mg mL^–1^) and muscle (GlucoMuscle, mg mL^–1^), and caloric content of muscle (CaloricCont, cal g^–1^). The total wet weight, hepatopancreas and gonad weight (g) were obtained to calculate the hepatosomatic index (HSI,%) and the gonadosomatic index (GSI,%).

Non-metric multidimensional scaling was used to obtain configuration maps with reduced dimensions that would order and separate samples considering differences in all 14 descriptors simultaneously. This was achieved by calculating Gower’s dissimilarity index between all pairs of samples (*n* = 154; Legendre and Legendre, 1998). A permutational MANOVA (Anderson, 2001) was then used to examine variations in these descriptors amongst octopus combining sex and season of collection. The underlying experimental design was a two-way model with sex (2 levels) and season (3 levels). Sampling stations (3 levels) were nested within each season. Octopus total weight was included in the model as a covariable, thus forcing the use of a Type I sum of squares for the partitioning of total variation. Octopus total weight was included in the model as a covariable, thus forcing the use of a Type I sum of squares for the partitioning of total variation. A maximum of 9,999 unrestricted permutations of raw data were used to obtain the empirical distribution of *pseudo-F* values (Anderson, 2001; McArdle and Anderson, 2001). Multivariate paired comparisons between centroids were applied following a similar procedure to calculate empirical *pseudo-t* values. Statistical analyses were performed using PRIMER v6 plus PERMANOVA (Anderson et al., 2008).

## Results

The physicochemical characteristics of seawater at the bottom of capture sites were stable in salinity (35.4 to 36.4 PSU), pH (8.2 to 8.3), and dissolved oxygen (7.1 to 7.3 mg/L). The largest variation was recorded in seawater temperature from 25°C in the winter to 27°C in the dry season and 28°C in the rainy season (Table 1).

**TABLE 1:** Temperature, salinity, dissolved oxygen and pH of seawater samples taken at the collecting sites (8 m depth) of *Octopus maya* in each season.

Month	Winter, February	Dry, May	Rainy, September
Temperature, °C	25 ± 0.5	27 ± 0.6	28 ± 0.7
Salinity, PSU	36.4 ± 0.1	35.3 ± 0.1	36.3 ± 0.1
Dissolved oxygen, mg mL^–1^	7.08 ± 0.1	7.21 ± 0.1	7.30 ± 0.1
pH	8.2 ± 0.1	8.26 ± 0.1	8.24 ± 0.1

*Values are mean ± SE.*

The ratio of female and male specimens caught was consistent with the reproductive dynamics of *O. maya*: during the rainy season the ratio was 1:1.1 and changed to 1:1.3 and 1:1.4 during the dry and winter storm seasons, reflecting a lower proportion of females due to parental care and/or death after hatching.

The 154 *O. maya* comprised 87 males and 67 females. Wet weight of specimens varied strongly among seasons (from 131 to 1023 g). Octopus with the lowest total weights were recorded in the rainy season (392 ± 20 g), followed by those collected in the winter (447 ± 24 g). The largest specimens were caught in the dry season (535 ± 23 g).

**FIGURE 1:**
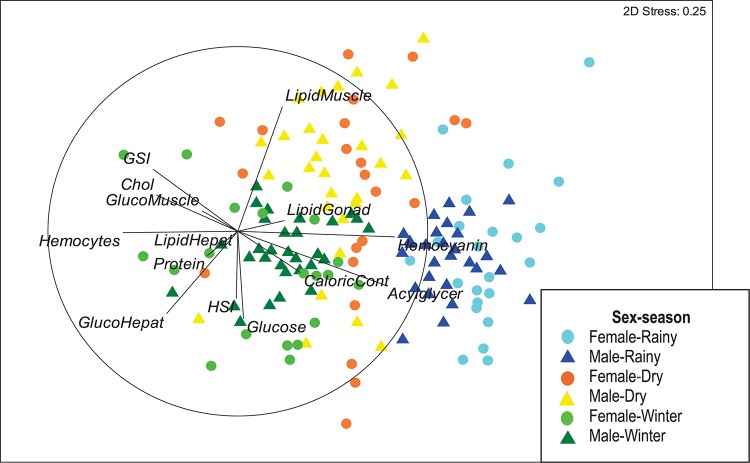
Non-metric Multidimensional Scaling (NMDS) of 14 descriptors biochemical composition: hemolymph (hemocyanin, hemocyte concentration, total protein, glucose, cholesterol and acylglycerides), gonads, hepatopancreas and muscle (glycogen concentration and total lipids), caloric value of muscle, hepatosomatic index (HSI) and gonadosomatic index (GSI) in *Octopus maya* caught in three seasons (winter, dry, and rainy season).

The 2-dimensional NMDS showed an effective separation of samples according to the season in which octopus were collected (Figure 1). The horizontal axis ordered samples with the highest values of hemocyanin and acylglycerids in right hand side corresponding to individuals collected during the rainy season (light and dark blue). These samples however, were low in hemocytes, cholesterol and GSI. By contrast, samples from individuals collected during the winter season were located in the lower left of the configuration map (light and dark green) and had high hemocyte count, glucose and glucose in hepatopancreas, but relatively low values of hemocyanin and acylglicerids. Octopus collected during the dry season (light and dark orange) were located in the upper half of the configuration map, corresponding to samples with high lipid content in muscle, but low glucose, glucose in hepatopancreas and HSI. Samples from female octopus were consistently more disperse than those from males (Figure 1). A stress value of 0.25 was obtained with the 2-D plot configuration, indicating a sufficiently adequate configuration that requires caution in graphical interpretation.

The permutational MANOVA (Table 2) showed that differences between the season in which octopus were collected contributed significantly to explain the variation in biochemical composition of the tissues sampled (*pseudo-F* = 7.3; *p* < 0.001). It also showed significant differences in biochemical descriptors between male and female octopus (*pseudo-F* = 6.1; *p* < 0.05). However, the non-significant interaction term indicated that the biochemical composition changed with the seasons in a similar way regardless of sex (*pseudo-F* = 1.3; *p* = 0.279). Further multivariate pairwise comparisons revealed significant differences between all three seasons (Table 3), thereby statistically confirming the graphical separation of samples previously described. The analysis also showed a significant contribution of total weight as a covariate in the model (*pseudo-F* = 8.6; *p* < 0.001). This result indicates there was considerable variation in the biochemical descriptors that can be attributed to octopus biomass, and suggests that total weight should be included in the analyses in order to unmistakably distinguish changes amongst seasons. Differences between sampling stations nested within each season were also significant (*pseudo-F* = 5.4; *p* < 0.001), but had no influence on the main trends detected. Table 4 shows the hemocytes concentration and biochemical descriptors of tissues (hemolymph, hepatopancreas, gonads, and muscle) of *O. maya* caught in three seasons (winter, dry, and rainy season).

**TABLE 2:** Results of the permutational MANOVA applied to hemocyte concentration and the biochemical composition of tissues (hemolymph, hepatopancreas, gonads, and muscle) of *Octopus maya* caught in three seasons (winter, dry and rainy season).

Source	df	SS	MS	*Pseudo*	*P*	Unique
				-*F*	(perm)	permutations
Total weight	1	2375	2375	8.638	0.0001	9932
Season	2	10122	5061	7.265	0.0001	9788
Sex	1	1298	1298	6.086	0.0106	9948
Sampling (Season)	6	4259	709	5.374	0.0001	9850
Season x Sex	2	458	229	1.339	0.2797	9941
Sex x Sampling	6	1009	168	1.273	0.1167	9877
(Season)						
Residuals	133	17568	132			
Total	150	37091				

*Degrees of freedom (df); sum of squares (SS); Mean square (MS); *pseudo-F* value obtained with a permutational MANOVA; *P (perm)* proportion of *F* values smaller than the *pseudo-F* value obtained with permutations; number of unique permutations used to calculate previous values.*

**TABLE 3:** Multivariate pairwise comparisons amongst centroids calculated based on hemocyte concentration and biochemical descriptors measured in the tissues (hemolymph, hepatopancreas, gonads, and muscle) of *Octopus maya* collected in three seasons (winter, dry, and rainy season).

Pairwise comparisons	*pseudo-F*	*P* (perm)	Unique permutations
Dry *vs.* Rainy	2.8	<0.001	720
Dry *vs.* Winter	1.8	<0.01	719
Rainy *vs.* Winter	3.5	<0.001	718

**pseudo-F* value obtained with a permutational MANOVA; *P (perm)* proportion of *F* values smaller than the *pseudo-F* value obtained with permutations; number of unique permutations used to calculate previous values.*

**TABLE 4:** Hemocytes concentration and biochemical composition (mean ± standard error) of tissues (hemolymph, hepatopancreas, gonads, and muscle) of *Octopus maya* caught in three seasons (winter, dry, and rainy season).

	Winter		Dry		Rainy	
	Females	Males	Females	Males	Females	Males
Number	21	30	22	29	24	28
Total wet weight, g	438 ± 43	473 ± 34	511 ± 29	557 ± 34	360 ± 27	407 ± 30
Hepatosomatic index (HSI,%)	3.71 ± 0.21	2.59 ± 0.12	2.58 ± 1.14	2.12 ± 0.09	2.97 ± 0.09	2.91 ± 0.08
Gonadosomatic index (GSI,%)	1.70 ± 0.57	0.75 ± 0.05	1.39 ± 0.43	0.72 ± 0.21	0.35 ± 0.17	0.56 ± 0.05
Hemocyanin, mM L^–1^	1.69 ± 0.06	1.80 ± 0.06	2.03 ± 0.05	2.06 ± 0.05	2.73 ± 0.05	2.69 ± 0.05
Hemocytes, cells mm^–3^	10567 ± 2072	18329 ± 2203	12501 ± 2088	18289 ± 2165	7030 ± 1201	8404 ± 1615
Proteins, mg mL^–1^	141 ± 11	152 ± 7	111 ± 14	141 ± 10	119 ± 9	101 ± 15
Cholesterol, mg mL^–1^	0.025 ± 0.001	0.026 ± 0.001	0.050 ± 0.005	0.048 ± 0.005	0.014 ± 0.001	0.016 ± 0.003
Glucose, mg mL^–1^	0.122 ± 0.01	0.125 ± 0.01	0.119 ± 0.01	0.106 ± 0.01	0.115 ± 0.01	0.114 ± 0.01
Acylglycerides, mg mL^–1^	0.035 ± 0.001	0.037 ± 0.001	0.036 ± 0.005	0.033 ± 0.003	0.080 ± 0.003	0.082 ± 0.001
Lipids in hepatopancreas, mg mL^–1^	42.99 ± 2.77	42.61 ± 1.76	37.84 ± 1.37	42.37 ± 2.08	40.97 ± 2.45	43.17 ± 1.94
Lipids in gonads, mg mL^–1^	24.03 ± 1.81	20.87 ± 1.16	32.60 ± 2.70	29.48 ± 2.11	32.21 ± 1.71	22.86 ± 0.95
Lipids in muscle, mg mL^–1^	5.66 ± 0.41	5.33 ± 0.35	9.56 ± 0.73	9.71 ± 0.66	8.50 ± 0.73	7.00 ± 0.37
Glycogen in hepatopancreas, mg mL^–1^	4.30 ± 0.40	4.04 ± 0.25	4.07 ± 0.43	3.44 ± 0.34	3.49 ± 0.23	3.22 ± 0.12
Glycogen in gonads, mg mL^–1^	3.42 ± 0.50	0.77 ± 0.08	2.98 ± 0.66	1.87 ± 0.62	1.42 ± 0.48	0.58 ± 0.17
Glycogen in muscle, mg mL^–1^	0.41 ± 0.08	0.25 ± 0.02	0.28 ± 0.02	0.25 ± 0.01	0.27 ± 0.03	0.25 ± 0.02
Caloric content muscle, g^–1^cal	4373 ± 124	4421 ± 73	4653 ± 88	4355 ± 51	4566 ± 51	4601 ± 48

## Discussion

The effects of climatic season and sex on the biochemical composition of *O. maya* were assessed for the first time. The gonadosomatic index (GSI) has been widely used to estimate the stage of sexual maturity and to determine the reproductive dynamics in cephalopods belonging to the family Octopodidae (Mangold, 1983). The highest GSI values were recorded in organisms caught in the dry and winter seasons, which is consistent with the reproductive period. According to the gonad maturation stages and to variations in progesterone and testosterone levels in the gonad of *O. maya*, the reproductive season occurred from February to June (Avila-Poveda et al., 2015). GSI in *O. maya* increased progressively concurring with the reproductive period. The same pattern was reported by Zamora and Olivares (2004) in female *Octopus mimus*, De Moreno et al. (1998) in *I. argentinus*, and Otero et al. (2007) in *O. vulgaris*. GSI in *O. maya* males varied less between seasons (0.75, 0.72, and 0.56% in winter, dry and rainy season respectively), indicating reproductive readiness from 160 to 1000 g. Considering that its lifecycle is short and dies after breeding, male maturity throughout the year represents an ecological advantage allowing more opportunities for reproduction. This behavior has also been reported in *O. mimus* males (Ishiyama et al., 1999) and in *O. vulgaris* in the Mediterranean Sea and in the eastern Atlantic Ocean (Mangold, 1983).

Environmental factors have a strong impact on animal reproduction; among these, light has been signaled out as the main factor involved in the reproductive activities of different species (Zúñiga-Romero et al., 1995). *O. vulgaris* size and the age at sexual maturity seems to depend primarily on light, temperature, and feeding (Mangold, 1983; Forsythe, 1993). Reserves have a strong influence on sexual maturation, leading to nutrient mobilization and accumulation. The present results showed a distinct seasonal variation in the biochemical composition of *O. maya* tissues. The digestive gland stored more lipid and glycogen than the gonad and muscle. This has also been reported in other cephalopods (Boucaud-Camou and Yim, 1980; Rosa et al., 2004, 2005; Sieiro et al., 2006). Lipid concentration in the digestive gland could be required for production and maturation of gametes, as well as during the egg-caring period, when females stop feeding. A recent research in post-spawning *O. maya* females reported the loss of 40% in wet weight during the incubation period, with higher weight loss in the gonad and hepatopancreas (Roumbedakis et al., 2017).

During the winter storm and the dry seasons, *O. maya* gonadal glycogen increased, possibly associated with the organisms’ response to the high demand of polysaccharides for the metabolic processes at the final phase of oogenesis and during the eggs caring period, which would be consistent with the species functional maturity. Carbohydrates are intermediary metabolic precursors for energy and non-essential amino acid production, and are components of ovary pigments (Harrison, 1990). These findings were also reported in *O. vulgaris* gonads (Rosa et al., 2004). The percentage of reproductively mature females and males is higher in the winter storm season than in the dry and rainy seasons, when the sea-surface temperature oscillates around 25°C (Angeles-González et al., 2017).

Higher values of lipid in muscle were recorded in the organisms caught in the dry season, which were the largest organisms captured, and therefore size could be associated with an increase in food intake to comply with the metabolic demands of growth. Similar results were reported in male and female *O. tehuelchus* muscle at intermediate maturation developmental stage (Pollero and Iribarne, 1988). These results are consistent with those reported by Rosa et al. (2005), for the muscles of the squids *Illex coindetii* and *Todaropsis eblanae* during the reproductive period.

The caloric value of muscle in both sexes increased during the rainy season, which could reflect a higher intake and/or provision of food prior to the reproductive period. In the winter season the caloric value decreased, possibly associated to the reproductive effort. Sexual maturation and breeding are the most energy-intensive investment periods of cephalopods lifecycle (Rosa et al., 2004). During the periods of catabolism and high energy demand, tissue proteins are mobilized to provide energy (Castro et al., 1993; Jackson and Mladenov, 1994).

Although the biochemical composition of the digestive gland, gonad, and muscle has been well studied in cephalopods, analysis of hemolymph components is still poorly known. This work provides a more comprehensive approach on the seasonal dynamic of cephalopods reserves mobilization and use. *O. maya* hemocyanin concentration has distinct seasonal variations, decreasing during the winter and dry seasons when reproduction takes place. Cholesterol is an important precursor of vitamin D and steroid hormones, a structural component of cellular membranes and precursor of sexual hormones involved in reproductive control (Kanazawa, 2001). The present study found that cholesterol concentration in hemolymph was higher in the largest organisms caught in the winter and dry seasons, coincident with the main reproductive period (Avila-Poveda et al., 2015; Markaida et al., 2017). Heras and Pollero (1989) also reported cholesterol variations in plasma associated with *O. tehuelchus* sexual maturation. Cephalopods are known to have a low capacity to synthetize sterols (Voogt, 1973; Goad, 1978), therefore cholesterol comes mainly from the diet (Rosa et al., 2004).

In crustaceans, hemocyanin is used as energy substrate and amino acid source in situations of high energy demand and starvation periods (Rosas et al., 2002; Pascual et al., 2003). In cephalopods, hemocyanin is probably involved as a source of metabolic energy. The present results indicate that hemocyanin could be associated with protein transfer for sexual maturation, which concurs with the highest values of GSI. Protein concentration in plasma did not change with seasons, but displayed an opposite pattern to hemocyanin concentration, which could reflect the unfolding of the hemocyanin molecule and release of proteins. These proteins could be used as energy substrate and as source of amino acids for gametes production. They are also structural components of tissues and can be used as reserves at the final development stages.

The decrease in *O. maya* plasma acylglycerides concentration at the pre-reproductive period may reflect lipid mobilization, transported by hemolymph primarily to gonadal tissue. Hemocyanin, a lipoprotein, has an important role in lipid transport (Heras and Pollero, 1990). Heras and Pollero (1989) found plasma acylglycerides variations associated with sexual maturation of *O. tehuelchus*. They also determined the lipid and fatty acid composition of hemocytes and plasma at different sexual development stages. These authors reported that the highest concentrations of lipids linked to eggs development were constituted by esters, triacylglycerols and diacylglycerols. Heras and Pollero (1990, 1992), detected the presence of three lipoproteins that transport mainly cholesterol and phospholipids.

The pattern observed in metabolites and reserves indicates a seasonal variation associated with the growth of the organisms and the reproductive peak, but it may also reflect the physiological response to seawater temperature. Studies on reproductive conditions of *O. maya* suggest that variations in population parameters could be linked to the geographic distribution of thermal zones (Angeles-González et al., 2017). Low values of hemocyanin, acylglycerides, lipids, and glycogen concentrations could possibly reflect the metabolic cost of being exposed to high temperatures. High temperatures (28 to 31°C) have been demonstrated to affect *O. maya* reproductive capability by inhibiting spawning (Juárez et al., 2015), compromising male maturation (López-Galindo et al., 2018), health status (Pascual et al., 2019) and affecting the viability of eggs and progeny growth (Sánchez-García et al., 2017). Further studies on the physiological condition of wild organisms are required to better understand the relation between nutritional status and environmental variations. Improving our knowledge on cephalopods response to temperature is highly relevant in the wake of a global warming scenario. Monitoring the environmental conditions in ecosystems with important commercial species to anticipate relevant changes has been widely recommended. This is particularly important in thermosensitive species narrowly distributed, such as *O. maya.*

The present study complements the baseline information on this species. It provides reference values of several physiological indicators for *O. maya* sub-adult and adult organisms that may be useful for monitoring programs of wild populations, as well as to design diets and management protocols to produce octopus under controlled conditions. Nowadays there is a huge interest in establishing cephalopods aquaculture and a better understanding of nutrient use and mobilization is relevant to advance knowledge on energy demand during sexual maturation and reproductive activity.

## Data Availability Statement

The datasets generated for this study are available on request to the corresponding author.

## Ethics Statement

The Mexican oficial norm (NOM-062-ZOO-1999) on the technical specifications for the production, use and care of laboratory animals does not include marine invertebrates, and regulations on the matter are scarce. We followed the Guide for the Care and Use of Experimental Animals in Research and Teaching of the National Aotonomous University of Mexico.

## Author Contributions

CP, CR, MM, and PG conceived and designed the study. CP, HC-L, and AS conducted the experimental procedures. MM and CP analyzed and interpreted the data. MM, CP, HC-L, PG, and PD wrote the original draft.

## Conflict of Interest

The authors declare that the research was conducted in the absence of any commercial or financial relationships that could be construed as a potential conflict of interest.
